# In vitro activity of cefiderocol against carbapenem-resistant *Acinetobacter baumannii* carrying various β-lactamase encoding genes

**DOI:** 10.1007/s10096-024-04831-w

**Published:** 2024-04-23

**Authors:** Aylin Uskudar-Guclu, Salih Danyildiz, Hasan Cenk Mirza, Mehtap Akcil Ok, Ahmet Basustaoglu

**Affiliations:** 1 Department of Medical Microbiology, Baskent University Faculty of Medicine, Ankara, Türkiye; 2 Department of Nutrition and Dietetics, Baskent University Faculty of Health Sciences, Ankara, Türkiye

**Keywords:** Cefiderocol, MDR *Acinetobacter baumannii*, Multidrug Resistance, EUCAST, CLSI

## Abstract

**Objectives:**

This study aimed to determine the in vitro efficacy of cefiderocol in carbapenem-resistant *Acinetobacter baumannii* (CRAB) isolates and evaluate the disk-diffusion (DD) method as an alternative method to broth-microdilution (BMD).

**Methods:**

Totally 89 CRAB isolates were included. Cluster analysis was determined by Pulsed-Field Gel Electrophoresis (PFGE). Resistance genes; *bla*_OXA−51_, *bla*_OXA−23_, *bla*_OXA−24_, *bla*_OXA−58,_*bla*_PER−1_, *bla*_NDM_, *bla*_IMP_ and *mcr-1* were screened. Cefiderocol susceptibility testing was performed by both DD and BMD. Interpretation was made according to EUCAST and CLSI. Categorical agreement (CA), minor errors (mEs), major errors (MEs), and very major errors (VMEs) were determined.

**Results:**

PFGE revealed 5 distinct pulsotypes; 86 of the isolates were extensively drug-resistant (XDR). All the isolates were negative for *bla*_NDM_, *bla*_IMP_, *mcr-1*, while positive for *bla*_OXA−58_ and *bla*_OXA51_. *bla*_PER−1_ was positive for 33.7%; *bla*_OXA−23_ for 74.2%; *bla*_OXA−24_ for 12.3%. According to CLSI, the MEs rate was 1.85%, mEs was 7.86% and there were no VMEs. According to EUCAST, MEs rate was 3.70%, there were no mEs and VMEs. CA was 91% for CLSI and 97.8% for EUCAST. MICs of cefiderocol against *A. baumannii* isolates ranged from 0.06 to > 128 mg/L, with MIC50 and MIC90 values of 0.5 and > 128 mg/L, respectively.

**Conclusions:**

Cefiderocol susceptibility was 60.7% in CRAB isolates. MIC50, MIC90 of *bla*_PER−1_ positive and *bla*_PER−1_ negative groups were > 128/>128 and 0.25/>128 mg/L. A correlation between the presence of *bla*_PER−1_ and cefiderocol resistance was observed (*p* < 0.0001). Among colistin-resistant isolates, the presence of *bla*_PER−1_ was 47.1% and 75% of them were resistant to cefiderocol respectively.

**Supplementary Information:**

The online version contains supplementary material available at 10.1007/s10096-024-04831-w.

## Introduction

Antimicrobial resistance (AMR) is one of the most serious worldwide public health threats that poses significant challenges to healthcare systems including mortality, morbidity, and healthcare costs [1]. It was estimated that about 5 million deaths were associated with bacterial AMR in 2019 [2]. World Health Organization (WHO) published a list of antibiotic-resistant priority bacterial pathogens that pose the greatest threat to human health, in which carbapenem-resistant *A. baumannii* (CRAB) was declared as “*critical*” [3]. CRAB has emerged as a leading cause of healthcare-associated infections, such as pneumonia, bloodstream infections, and urinary tract infections particularly in immune-compromised individuals. A key aspect of *A. baumannii* is the propensity to develop rapid resistance [4]. According to The Central Asian and European Surveillance of Antimicrobial Resistance network [1], the carbapenem resistance rate of *A. baumannii* exceeded 90% in some countries such as Turkiye, Serbia, Belarus, and Montenegro. Carbapenem resistance in *A. baumannii* is mediated by multiple different mechanisms but mostly by acquisition of Ambler class D enzymes, in particular, OXA-23, and OXA-24 [5, 6]. Other carbapenemases, such as OXA-58, NDM, KPC, and IMP have also been reported [6, 7]. Also, extended-spectrum β-lactamases (ESBL) including PER-type which efficiently hydrolyzes penicillins and cephalosporins [8], contribute the emergence of widespread multi-drug-resistant (MDR) strains of *A. baumannii.* This situation has forced clinicians to use colistin as one of the last therapeutic options in the fight against such infections [9, 10]. According to WHO’s 2020 Global Antimicrobial Resistance Surveillance System data, the rate of colistin resistance worldwide reaches 37% in some countries such as Lithuania. In a study conducted in Turkiye, *A. baumannii* was reported as the most common isolate (25.1%) of bacterial lower respiratory tract (LRT) infection and the most effective antibiotic was colistin with a resistance rate of 12.9% [11]. The mechanisms of colistin resistance may be due to chromosomal mutations (such as *lpxACD*, *pmrB* and *pmrA*) or transferable plasmid genes called *mcr*, which can be spread between bacteria by horizontal transfer [12]. With the emergence of resistant isolates, last resort carbapenem and lipopeptide class antibiotics are no longer effective, highlighting the urgent need for new and effective treatment options.

Cefiderocol is a newly developed siderophore cephalosporin [13]. Like other β-lactam antibiotics, it binds to penicillin-binding proteins (especially PBP-3) and has a bactericidal effect on bacterial cell wall synthesis [14, 15]. What makes it unique is its increased transition to the bacterial periplasmic area by iron transport systems with its siderophore-like property [14, 15]. It owes this feature to the catechol part, which is located in the 3rd position of the side chain in its molecular structure and binds to ferric iron [13, 16]. It shows increased stability against β-lactamases due to the catechol group [14–16]. Although various mechanisms including amino acid changes in PBP-3, changes in the transcription levels of iron transport protein-encoding genes such as *fiu*, *feoA*, and *feoB*, and downregulation in the TonB-ExbB-ExbD energy transfer system cause cefiderocol resistance in *A. baumannii* [17–19], it is emphasized that some β-lactamases contribute to cefiderocol resistance [17, 19]. PiuA, a TonB-dependent siderophore receptor, and PirA, a ferric-enterobactin receptor, are very important in cefiderocol uptake [19–21]. In various studies, disruptions in the *piuA* gene (such as a frameshift mutation resulting in stop codon formation (K384fs) and F174Y substitution) or in the *fepA* gene, an ortholog of the *pirA* gene, (such as a transposon insertion, P635-ISAba125) caused increases in cefiderocol MICs ​​and resistance [20–23]. In another study, *piuA* and *pirA* gene deletion mutants showed increased MICs ​​against siderophore-drug conjugates compared to the standard strain without the deletion [22]. PER-1 as an Ambler class A ESBL, NDM as an Ambler class B metallo- β-lactamase, and OXA-23, OXA-24, and OXA-58 as Ambler class D carbapenemases have been reported among important β-lactamases to cefiderocol resistance [17, 18, 24].

In this study, it was aimed to evaluate the in vitro efficacy of cefiderocol in CRAB isolates with a various resistance gene profile. Broth-microdilution (BMD) is recommended as the reference standard method for cefiderocol susceptibility testing [25, 26], and iron depleted-cation adjusted Mueller Hinton broth (ID-CAMHB) should be used which is not available as ready-to-use. It was also aimed to evaluate disk-diffusion (DD) as an alternative to the BMD.

## Material and method

This study was approved by the Baskent University Institutional Review Board (Project no: KA22/392) and supported by Baskent University Research Fund.

### Bacterial isolates

A total of 89 CRAB isolates from the culture collection of Medical Microbiology Laboratory of Baskent University Medical Faculty collected between January to December 2021 were included in the study. The 32.6% (*n* = 29) of the collected isolates were obtained from lower respiratory tract samples, 23.6% (*n* = 21) from blood, 20.2% (*n* = 18) from urine,13.5% (*n* = 12) from wound, 5.6% (*n* = 5) from catheter, and 4.5% (*n* = 4) from biopsy culture. Only one isolate for each patient was included. Identification and antimicrobial susceptibility testing (AST) were performed by the BD Phoenix 50 (BD Diagnostics, USA) automated bacterial identification and antibiotic susceptibility system. The identification was confirmed by the presence of the chromosomal *bla*_OXA−51_ gene by PCR. All isolates were resistant to meropenem and imipenem, resistance was also screened by DD. MICs of colistin were determined by the Thermo Fisher Scientific Sensitivity FRCOL kit (Thermo Fisher Scientific, USA). All the AST results were assessed using EUCAST standards [27].

### Cluster analysis

Genetic relations among clinical CRAB isolates were determined by PFGE as previously described [28]. Genomic DNA was treated with lysis solutions and digested with ApaI restriction enzyme (Takara, Japan). Electrophoresis was performed in a PFGE system (CHEF-DR III System; Bio-RAD, USD). The gel images were processed and cluster analysis was performed using BioNumerics software. The images were normalized by using standard molecular markers, and banding patterns were compared. Pulsotype typing was performed by the interpretative criteria of Tenover et al. [29].

### Detection of antibiotic resistance genes by PCR

Bacterial DNA was extracted by boiling the bacterial suspension at 95^o^C for 10 min [30]. After centrifugation, the supernatant was transferred to new tubes. Amplification of OXA genes, *bla*_OXA−51_, *bla*_OXA−23_, *bla*_OXA−24_, and *bla*_OXA−58_ were performed as previously described [31] The *bla*_PER−1_, *bla*_NDM_, *bla*_IMP,_ and *mcr-1* genes were screened as described previously [32–34]. The primer sequences and amplicon sizes were given in supplementary material. Confirmed positive strains of *A. baumannii* carrying *bla*_OXA51_ gene [35], *K. pneumoniae* CDC529 carrying *bla*_NDM−1_, *K. pneumoniae* CDC309 *bla*_IMP_, and *A. baumannii* ATCC19606 were used as positive control strains. The PCR products were analyzed by 2% agarose gel containing EtBr.

### Cefiderocol susceptibility detection by Kirby Bauer DD method

Cefiderocol susceptibility testing was performed by using MASTDISCS (30 µg) (Mast Group, UK) and cation-adjusted Mueller Hinton agar (CA-MHA) (Oxoid, UK). Plates were incubated for 18–24 h at 35 ± 2 °C in aerobic conditions. Zone diameters were evaluated in the reflected light and from the back of the plate. Interpretations of zone diameters were made according to both EUCAST and CLSI [27, 36].

### Cefiderocol MIC detection by BMD

MIC detection was performed by using ready-to-use broth microdilution plates, ComASP®, available in the range of cefiderocol 0.008-128 mg/L (Liofilchem, Italy) according to the recommendation of the manufacturer. Briefly, a suspension of 0.5 McFarland standard was prepared from fresh bacterial colonies grown in overnight incubation. This prepared suspension was diluted 1:20 in 0.9% NaCl. 0.4 ml of the diluted bacterial suspension was added to the tube containing 3.6 ml of ID-CAMHB supplied in the kit. From this suspension, 100 µl were distributed into each well, containing cefiderocol at different concentrations between 0.008 mg/L to 128 mg/L. The plates were incubated for 16–20 h at 35°±2 °C in aerobic conditions. The first well in which the turbidity first disappeared or the turbidity showed a significant decrease compared to the growth control well due to the “trailing effect” was considered as the MIC value. When the “Trailing effect” was observed, 80% inhibition was accepted as the MIC value [25]. MIC value was evaluated in the reflected light from the front of the sensititre plate. Interpretation was made according to both EUCAST and CLSI criteria [27, 36]. *E. coli* ATCC 25922 and *P. aeruginosa* ATCC 27853 were used as quality control strains for both methods.

### Agreement analysis

By using the BMD as a reference method; categorical agreement (CA), minor errors (mEs), major errors (MEs), and very major errors (VMEs) were determined according to CLSI definitions. Acceptance criteria are based on ≥ 90% CA, ≤ 10% mEs, ≤ 3% MEs, and ≤ 1.5% VMEs [37].

### Statistical analysis

Pearson and Likelihood Ratio Chi-square test were used for the comparison of categorical variables between groups, and the significance test of the difference between two proportions was used for the comparison of only one result within two groups. Categorical variables were expressed as f (%). Statistical analysis was performed by using Statistical Package for Social Sciences (SPSS) for Windows Ver25.0. In statistical analysis, the significance level was taken as *p* < 0.05.

## Results

### Isolate profile

All isolates were collected between January to December 2021. The 86 of all isolates were XDR, and 3 were MDR. According to routinely performed AST results 92.1% (82/89) were resistant (R) to amikacin, 97.7% (87/89) to gentamicin, ciprofloxacin, and levofloxacin, 98.8% (88/89) to trimethoprim-sulfamethoxazole and 19.1% (17/89) to colistin. For tigecycline MIC distribution; 2/89 (2.2%) isolates MICs were 1 mg/L, 1/89 (1.1%) isolates < 2 mg/L, 10/89 (11.2%) isolates 2 mg/L and 76/89 (85.5%) isolates were > 2 mg/L.

All the isolates were found as negative for *bla*_NDM_, *bla*_IMP_, *mcr-1*, and *bla*_OXA−58_ while positive for *bla*_OXA51_. *bla*_PER−1_ was positive for 33.7% (30/89); *bla*_OXA−23_ was positive for 74.2% (66/89); *bla*_OXA−24_ was positive 12.3% (11/89) of the isolates.

PFGE revealed 5 distinct pulsotypes; one major, two intermediates, and two minors (Supplementary Fig. 1) [29]. The major pulsotype included 59 isolates (66.3%) and 96.8% (*n* = 56) of them were XDR. There were 14 (15.7%) and 9 (10.1%) isolates in intermediate pulsotypes and minor pulsotypes included 2 (2.3%) and 5 (5.6%) isolates.

### Susceptibility of cefiderocol by DD and BMD

According to CLSI [36], cefiderocol MIC value of ≤ 4 mg/L in *A. baumannii* is considered as S, 8 mg/L as I, and ≥ 16 mg/L as R; cefiderocol zone diameter of ≥ 15 mm in *A. baumannii* is considered as susceptible (S), 11–14 mm as intermediate (I), and ≤ 10 mm as R. There is no I category in EUCAST, and resistance of *A. baumannii* based on PK/PD values while recommending breakpoints as ≤ 2 mg/L S and > 2 mg/L as R; cefiderocol zone diameter ≥ 17 mm as S [27]. MICs of cefiderocol against *A. baumannii* isolates ranged from 0.06 to > 128 mg/L, with MIC50 and MIC90 values of 0.5 and > 128 mg/L, respectively. The results of cefiderocol susceptibility test according to both EUCAST and CLSI were given in Table 1.

**Table 1:** Cefiderocol susceptibility results according to EUCAST and CLSI.

	Broth Microdilution		Disc Diffusion	
	CLSI	EUCAST	CLSI	EUCAST
**S**	60.7 (*n* = 54)	59.6 (*n* = 53)	59.6 (*n* = 53)	57.3 (*n* = 51)
**I**	0 (*n* = 0)	No I category	7.8 (*n* = 7)	No I category
**R**	39.3 (*n* = 35)	40.4 (*n* = 36)	32.6 (*n* = 29)	42.7 (*n* = 38)

### Correlation of DD method with BMD

According to CLSI, 1 R isolate (1.85%) (zone diameter 6 mm) by DD was found as S by BMD (MIC: 0.5 mg/L) which indicates a major error. 7 I isolate (7.86%) (zone diameters 13,14,14,13,13,14,13 mm) by DD were found as R (MICs:16,16,128,32,16,16 and 16 mg/L respectively) by BMD which indicates a minor error. There was no very major error according to CLSI. The 81 (91%) isolates were in categorical agreement.

According to EUCAST, 2 R isolates (3.70%) (zone diameters 15 and 6 mm) by DD were found as S (MICs:2 and 0,5 mg/L) by BMD indicating MEs. There are no VMEs according to EUCAST. Also, there are no mEs due to lack of an I category in EUCAST. Totally 87 (97.8%) isolates were in CA. Table 2 shows the VME, ME, mE, and CA rates of the DD according to CLSI and EUCAST.

**Table 2:** VME, ME, mE and CA rates with disk diffusion test according to CLSI and EUCAST

	CLSI (%)	EUCAST (%)
**VME**	0	0
**ME**	1.85 (*n* = 1)	3.70 (*n* = 2)
**mE**	7.86 (*n* = 7)	0
**CA**	91.0 (*n* = 81)	97.75 (*n* = 87)

### In vitro cefiderocol activity according to gene profile

The contribution of *bla*_PER−1_, *bla*_OXA−23_, and *bla*_OXA−24_ genes to cefiderocol resistance was evaluated. Totally 30 (33.7%) isolates were positive for *bla*_PER−1_ gene and 25 (83.3%) *bla*_PER−1_ positive isolates were resistant to cefiderocol. While 10 (11.2%) of 59 *bla*_PER−1_ negative isolates were resistant to cefiderocol. MIC50 and MIC90 values of *bla*_PER−1_ positive and *bla*_PER−1_ negative isolates were > 128/>128 and 0,25/>128 mg/L respectively. Totally 66 (74.2%) isolates were *bla*_OXA−23_ positive and 28 (42.4%) of them were resistant to cefiderocol. While 7 (30.4%) of 23 *bla*_OXA−23_ negative isolates were resistant to cefiderocol. The 11 isolates were *bla*_OXA−24_ positive and 1 (9.1%) of them was resistant to cefiderocol while 34 (43.6%) *bla*_OXA−24_ negative isolates were resistant to cefiderocol. Table 3 shows the MIC distributions of isolates in the presence of resistance genes and Table 4 shows the susceptibility rates in the presence of resistance genes individually and in combinations. Cefiderocol activity of colistin-resistant isolates in the presence and absence of the *bla*_PER−1_ gene were also evaluated and shown in Table 5.

**Table 3:** MIC distributions of isolates in the presence of resistance genes

		MIC (mg/L)																		
**Ambler Class**	**Resistance Genes**	≤ 0.008	0.016	0.03		0.06		0.12	0.25		0.5	1	2	4	8	16	32	64	128	> 128
D	OXA-23 ( *n* = 66 )					2		10	7		11	1	6	1		3	4	1	4	16
D	OXA-24 ( *n* = 11 )							1	8		1								1	
A	PER-1 ( *n* = 30 )										2		3			2	3	1	4	15
**All isolates (** *n* ** = 89 )**						2		13	16		14	1	7	1		5	4	1	6	19

**Table 4:** Cefiderocol susceptibility rates in the presence of resistance genes individually and in combinations

Genes (n)	S (%)	I (%)	R (%)	p
PER-1 + (30)	5 (16.7)	0	25 (83.3)	< 0.0001
PER-1 – (59)	49 (83.1)	0	10 (16.9)	
OXA-23 + (66)	38 (57.6)	0	28 (42.4)	0.311
OXA-23 – (23)	16 (69.6)	0	7 (30.4)	
PER-1 + OXA-23+ (27)	5 (18.5)	0	22 (81.5)	< 0.0001
PER-1 + OXA-23 – (3)	0	0	3	
PER-1 - OXA-23 + (39)	33 (84.6)	0	6 (15.4)	
PER-1 - OXA-23 – (20)	16 (80.0)	0	4 (20)	
OXA-24 + (11)	10 (90.9)	0	1 (9.1)	0.062
OXA-24 – (78)	44 (56.4)	0	34 (43.6)	
PER-1 + OXA-24 + (0)	0	0	0	< 0.0001
PER-1 + OXA-24 – (30)	5 (16.6)	0	25 (83.4)	
PER-1- OXA-24 + (11)	10 (90.9)	0	1 (9.1)	
PER-1 - OXA-24 – (48)	39 (81.3)	0	9 (18.7)	
PER-1 + OXA-23 + OXA-24 + (0)	0	0	0	-
PER-1 - OXA-23 - OXA-24 – (11)	8 (72.7)	0	3 (27.3)	

**Table 5:** Colistin and cefiderocol susceptibilities of the isolates in the presence and absence of the PER-1

Isolates (n)	Cefiderocol Susceptibility (%)			
	S (n)	I (n)	R (n)	*p*
Colistin ( R ) (17)	64.7 (11)	0	35.3 (6)	0.674
Colistin ( S ) (72)	59.7 (43)	0	40.3 (29)	
Colistin ( S ) *bla*_PER−1_ + (22)	13.7 (3)	0	86.3 (19)	< 0.0001
Colistin ( S ) *bla*_PER−1_ – (50)	80.0 (40)	0	20.0 (10)	
Colistin ( R ) *bla*_PER−1_ + (8)	25.0 (2)	0	75.0 (6)	
Colistin ( R ) *bla*_PER−1_ – (9)	100 (9)	0	0	

## Discussion

Cefiderocol is a siderophore cephalosporin that possesses potent activity against carbapenem-resistant and MDR *Enterobacterales*, and non-fermentative Gram-negative bacilli [14, 16]. Cefiderocol received Food and Drug Administration (FDA) approval for the treatment of urinary tract infections in 2019 and treatment of hospital-acquired pneumonia and ventilator-associated pneumoniae in 2020. Although there are methods such as DD and gradient diffusion to test the susceptibility of cefiderocol, the gold standard method is the BMD [25, 26]. Standard cation-adjusted Mueller Hinton broth is not controlled for iron concentration and iron-depleted cation-adjusted MHB (ID-CAMHB)is required for susceptibility results to be accurate [26, 38]. The CLSI has approved ID-CAMHB and provides instruction to prepare for determining cefiderocol MICs but preparation of this medium is difficult and impractical for the daily laboratory workflow. Also, some gradient diffusion strips were developed but they were reported successful only in *P. aureginosa* [26]. For all these, DD was also tested for all the isolates included in the study as an alternative method in which standard CA-MHA can be used in the cefiderocol susceptibility testing.

If CLSI is considered in terms of compatibility of DD and BMD; the MEs was 1.85%; mEs was 7.86%, there was no VME while the CA was 91%. Acceptance criteria are based on ≥ 90% CA, ≤ 10% mEs, ≤ 3% MEs, and ≤ 1.5% VME [37]; therefore, the DD seems as a candidate to be a practical and easily applicable alternative to the BMD. But based on CLSI, a total of 7 isolates were in category I according to the DD, while all of these isolates were determined as R according to the BMD; so this result indicates that the results interpreted as I by DD might be R and it would be better to confirm them with the BMD.

According to EUCAST, the MEs was 3.70%, there was no mEs and VME while the CA was 97.8%. With an interpretation according to EUCAST, the DD does not seem as a candidate alternative to the BMD although the CA value was found to be higher than CLSI, the MEs rate (3.7%) was observed above the limit value (3.0%). This might be resulted from the lack of category I in EUCAST which ensures no mEs but causes more MEs.

In a study including CRAB isolates, DD was compared with BMD and HardyDisks with MASTDISCS, and CA was slightly higher in MASTDISCS than in HardyDisks [38]. They also found that for DD, EUCAST breakpoints demonstrated lower susceptibility at 66% compared to CLSI breakpoints with 89% for MASTDISCS. For MASTDISCS, EUCAST breakpoints resulted in the highest CA, however due to the lack of an I category, higher rates of MEs and VMEs were observed. They have indicated that both DD performed poor activity for *A. baumannii* complex. DD offers a convenient alternative approach to BMD at gram-negative bacteria for cefiderocol AST, with the exception of *A. baumannii* complex [38]. The results of this study also showed CLSI to be more appropriate than EUCAST as there is no I category [38]. Although DD was not reported as an alternative to BMD in *A. baumannii* complex in this study; according to our results DD can be an alternative to the BMD if CLSI is used for *A. baumannii*.

According to another study, 90.9% of 368 carbapenem non-susceptible *A. baumannii* isolates were found to be susceptible to cefiderocol, while the MIC90 value was 8 mg/L [39]. In another study covering different countries, 76.77% of MDR *A. baumannii* isolates remained susceptible to cefiderocol, and all cefiderocol resistant isolates were MDR [40]. In the present study, 60.7% of the isolates were S, 39.3% were R, 0% was I according to CLSI criteria; while 59.6% was S, and 40.4% was R according to EUCAST criteria. Higher resistance rates might have resulted from the fact that 96.6% of the isolates were XDR and the high prevalence of *bla*_PER−1_ gene (33.7%; *p* < 0.0001) in isolates may have contributed to cefiderocol resistance. The limitation of the study is that cefiderocol resistance mechanisms were not demonstrated by performing WGS on cefiderocol-susceptible and resistant isolates.

Cefiderocol is known for its high stability to β-lactamases, including ESBLs, AmpC, and carbapenemases [14–16]. But it was seen that the presence of *bla*_PER−1_ might result higher resistance rate than expected. Even in vitro activity may not reflect the clinical activity. In a study that compared the patient groups treated with the regimens containing cefiderocol to colistin, more microbiological failures were observed -especially in monotherapy- in cefiderocol treated group. All 46 *A. baumannii* isolates were found as sensitive in vitro according to CLSI and EUCAST. During treatment, microbiological failure was observed in 17.4% (8/46) of patients, and cefiderocol resistance was observed in 8.7% (4/46) [41].

Although many resistance mechanisms have been defined [18, 19, 21, 42], it is stated that the presence of certain β-lactamase genes might be a contributing factor in cefiderocol resistance. Among them, *bla*_PER−1_, *bla*_NDM_, *bla*_OXA−23_, *bla*_OXA−24_, *bla*_OXA−58_ are considered as very important factors [17, 18, 24, 43]. In our study, the presence of *bla*_PER−1_, *bla*_NDM_, *bla*_IMP_, *mcr − 1*, *bla*_OXA−23_, *bla*_OXA−24_, *bla*_OXA−51_, and *bla*_OXA−58_ genes are screened. The presence of *bla*_PER−1_ showed a significant contribution to cefiderocol resistance since 83.3% of *bla*_PER−1_ positive isolates were R to cefiderocol (*p* < 0.0001); while only 11.2% of *bla*_PER−1_ negative isolates were R. There was also significant difference between MIC50 values of *bla*_PER−1_ positive and *bla*_PER−1_ negative groups that MIC50 and MIC90 values were > 128/>128 and 0,25/>128 mg/L respectively. Some other studies also emphasized the role of the *bla*_PER−1_ gene in cefiderocol resistance [8, 17, 24, 44].

The cefiderocol resistance rate was higher in *bla*_OXA−23_ positive 28/66 isolates (42.4%) than *bla*_OXA−23_ negatives 7/23 (30.4%) but the difference was not statistically significant (*p* > 0.05). The contribution of *bla*_OXA−23_ has been emphasized in the co-existence of *bla*_OXA−23_ with other β-lactamase genes [18, 43]. The co-existence of *bla*_PER−1_ and *bla*_OXA−23_ rate was 30.4% in the current study and 81.5% of them were resistant to cefiderocol. The PER-1 plays a pivotal role in cefiderocol resistance. All *bla*_PER−1_ positive *bla*_OXA−23_ negative isolates (*n* = 3) were resistant to cefiderocol. Among *bla*_PER−1_ negative *bla*_OXA−23_ positive isolates (*n* = 39) the cefiderocol resistance rate was 15.4%. In isolates lacking both genes cefiderocol resistance rate was 20%. So, the presence of the *bla*_OXA−23_ gene seems to be only significant in association with the presence of *bla*_PER−1_ in cefiderocol resistance in *A. baumannii* (*p* < 0.0001). There was no statistically significant difference in cefiderocol resistance between the presence and absence of *bla*_OXA−24_ (*p* > 0.05). The co-existence of *bla*_PER−1_ and *bla*_OXA−24_ was not detected and among the isolates lacking both genes, cefiderocol resistance rate was significantly lower by 18.8% (*p* < 0.0001). There seemed to be no *bla*_OXA−24_ impact on cefiderocol resistance in *A. baumannii* isolates.

Colistin is the last resort for the treatment of MDR and XDR *A. baumannii* infections [9, 10] and the rate of colistin-resistant *A. baumannii* was observed as 12.9% in a study [11]. The mechanism of colistin resistance is due to chromosomal mutations (such as *lpxACD*, *pmrB*, and *pmrA*) or transferable plasmid genes called *mcr-1*, which can be spread between bacteria by horizontal transfer [12]. The 19.1% (17/89) isolates were resistant to colistin and none of the *A. baumannii* isolates was positive for plasmid-mediated *mcr-1* resistance. The whole genome sequence of one of our colistin-resistant isolates showed that the colistin resistance resulted from the mutation in *pmrA* gene as G75A, A120G, G192A, C405T, T450C, C463T and C633T (unpublished data). In another study, 8.2% of *A. baumannii* isolates were found to be resistant to colistin, and similar to our result, in *mcr-1-5* genes were not detected [45]. Cefiderocol was also declared as an alternate agent against colistin-resistant *A. baumannii* isolates and 64.7% (11/17) of the colistin resistant isolates and 59.7% (43/72) colistin-susceptible isolates were susceptible to cefiderocol which is similar (*p* > 0.05). The unexpected cefiderocol resistance rate (40.3%) in colistin-susceptible isolates again resulted from the presence of *bla*_PER−1_ gene was 30.6% (22/72), and 86.3% (19/22) of these isolates were resistant to cefiderocol. Among colistin-resistant isolates the presence of *bla*_PER−1_ was 47.1% (8/17), and 75% (6/8) of them were resistant to cefiderocol while all 9 *bla*_PER−1_ negative colistin R isolates were susceptible to cefiderocol (Table 5). The efficacy of cefiderocol against colistin-susceptible or resistant isolates is inversely proportional to the presence of *bla*_PER−1_. These results indicate that cefiderocol may be effective in colistin-resistant isolates; suggesting that the efficacy is related to the presence of the *bla*_PER−1_ gene rather than the colistin resistance status in *A. baumannii*.

### Electronic supplementary material

Below is the link to the electronic supplementary material.

Supplementary Material 1

## Data Availability

Not applicable.
