# Supplementary material for: In vitro activity of cefiderocol against carbapenem-resistant Acinetobacter baumannii carrying various β-lactamase encoding genes
Source: Eur J Clin Microbiol Infect Dis. 2024 Apr 23;43(6):1171–9. doi: 10.1007/s10096-024-04831-w (PMC11178621; doi:10.1007/s10096-024-04831-w)
Supplement: Supplementary file 1 — Supplementary Material 1 [file 10096_2024_4831_MOESM1_ESM.docx]

**Supplementary Material**

**
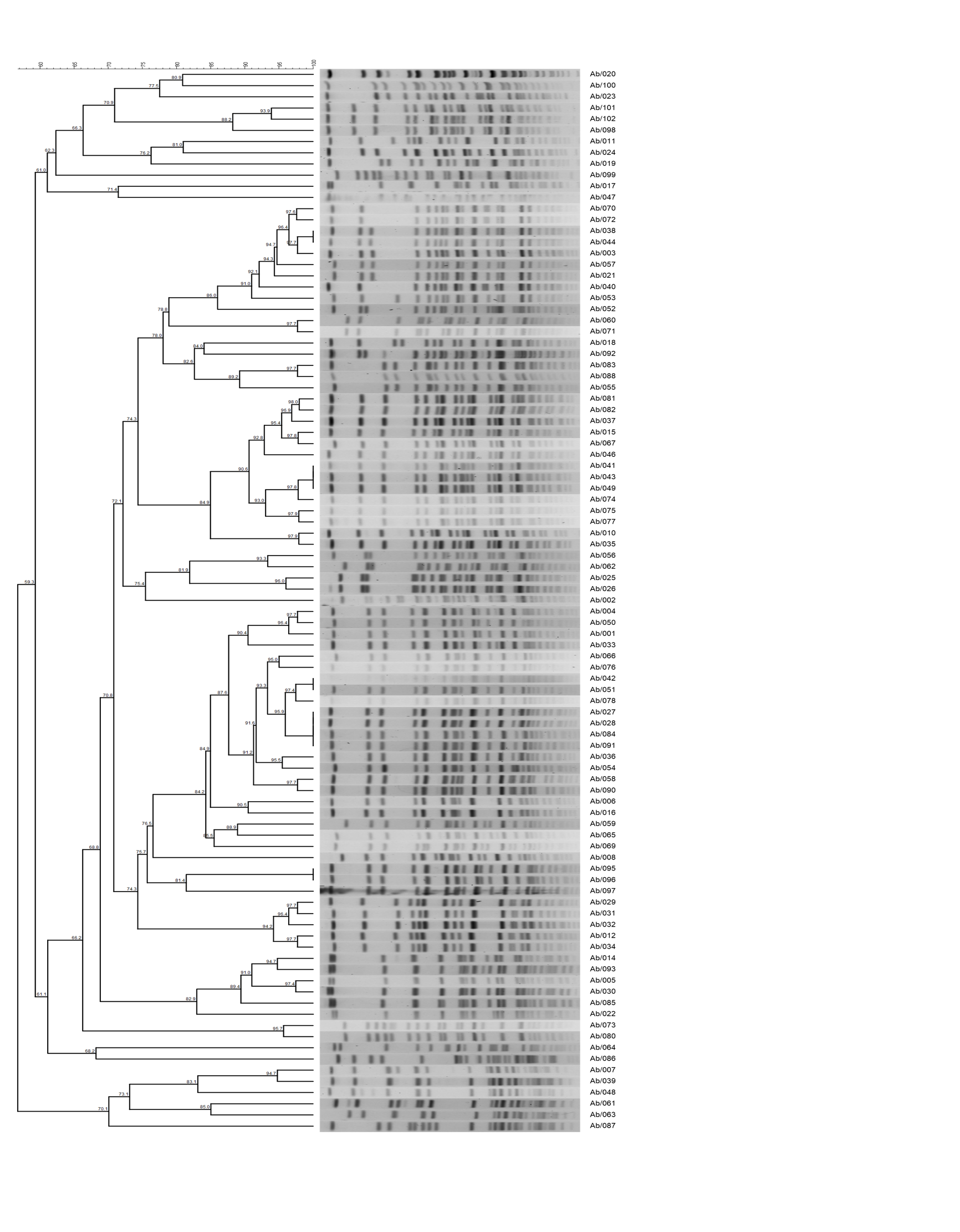
**

**Supplementary Figure 1:** PFGE dendrogram of CRAB isolates.

**Supplementary Table 1:** The primer sequences and amplicon sizes.

| **Genes** | **Primer sequence (5 →3)** | **Amplicon Size** |
| --- | --- | --- |
| *bla*_OXA-51_ | F-5’-TAATGCTTTGATCGGCCTTG-3’  R-5’-TGGATTGCACTTCATCTTGG-3’ | 353 bp |
| *bla*_OXA-23_ | F-5’-GATCGGATTGGAGAACCAGA-3’  R-5’-ATTTCTGACCGCATTTCCAT-3’ | 501 bp |
| *bla*_OXA-58_ | F-5’-AAGTATTGGGGCTTGTGCTG-3’  R-5’-CCCCTCTGCGCTCTACATAC-3’ | 599 bp |
| *bla*_OXA-24_ | F-5’-GGTTAGTTGGCCCCCTTAAA-3’  R-5’-AGTTGAGCGAAAAGGGGATT-3’ | 246 bp |
| *bla*_NDM_ | F-5’-GGTTTGGCGATCTGGTTTTC-3’  R-5’-CGGAATGGCTCATCACGATC-3’ | 621 bp |
| *bla*_IMP_ | F-5’-GGAATAGAGTGGCTTAAYTCTC-3’  R-5’-GGTTTAAYAAAACAACCACC-3’ | 232 bp |
| *bla*_PER-1_ | F-5’-AATTTGGGCTTAGGGCAGAA-3’  R-5-ATGAATGTCATTATAAAAGC-3’ | 925 bp |
| mcr-1 | F-5'-CGGTCAGTCCGTTTGTTC-3'  R-5'-CTTGGTCGGTCTGTAGGG-3' | 308 bp |
